# Interaction between dietary selenium intake and age on severe headache or migraine in the United States: a population-based study

**DOI:** 10.3389/fnut.2025.1537151

**Published:** 2025-03-25

**Authors:** Xinping Yu, Lanxiang Wu, Heqing Zheng, Wei Wu, Sheng Tian

**Affiliations:** ^1^Department of Neurology, The Second Affiliated Hospital, Jiangxi Medical College, Nanchang University, Nanchang, China; ^2^Institute of Neuroscience, Nanchang University, Nanchang, China

**Keywords:** migraine, dietary selenium intake, age, interactive effects, NHANES

## Abstract

**Background:**

Studies have shown that an antioxidant diet is a protective factor against migraine. However, the association between selenium, an important antioxidant consumed from the diet, and migraine has received little attention. The aim of this study was to explore the relationship between dietary selenium intake with migraine, with particular interest in age differences.

**Methods:**

This study based on cross-sectional data from people who took part in the National Health and Nutrition Examination Survey (NHANES) between 1999 and 2004. The multiple logistic regression model was applied to examine the association between selenium intake and migraine, and subgroup analyses were performed. Non-linear associations were explored with restricted cubic spline (RCS) models.

**Results:**

The study included a total of 9,849 adults aged 20 years and older. Compared with individuals with lowest selenium intake Q1 (≤59.4 ug/day), the adjusted OR values for selenium intake and migraine in Q2 (59.41–82.70 ug/day), Q3 (82.71–106 ug/day), Q4 (106.01–143.16 ug/day), and Q5 (≥143.17 ug/day) were 0.82 (95% CI: 0.64–1.05), 0.99 (95% CI: 0.77–1.26), 0.74 (95% CI: 0.54–0.99), and 0.68 (95% CI: 0.48–0.97), respectively. Sensitivity analyses showed a robust association between them. Our findings also suggested an interaction between age and selenium intake (*p* for interaction = 0.04). Additionally, the relationship between selenium intake and migraine in adults with 20–50 years was L-shaped. The OR of developing migraine was 0.97 (95% CI: 0.94–0.98) in individuals with selenium intake ≥101.9 ug/day in adults with 20–50 years.

**Conclusion:**

A higher dietary selenium intake is significantly associated with a decreased prevalence of migraine, and age can modify the association between them. Therefore, the present study indicate that an appropriate intake of selenium-rich foods in adults aged 20–50 years may prevent migraines.

## 1 Introduction

Migraine is a common neurological disorder that influences over 1 billion people worldwide and leaves approximately 45.1 million people with a long-term disability (1). Migraine is reported to be the leading cause of disability in adults under 50 years of age in terms of disability life expectancy (2). A number of studies have demonstrated gender differences in migraine. It is generally accepted that migraine is two to three times more common in females than males (3). Despite significant advances in the treatment of migraine, its pathophysiology remains unclear and the need for affordable, effective and safe methods of prevention remains high. Multiple medications are used to treat migraines, including triptans, ergots, anti-epilepsy drugs and antidepressants (4). However, these treatments, while effective, may lead to side effects such as dizziness, somnolence, fatigue, gastrointesyinal symptoms, and even cardiovascular disease (4). In recent years, complementary therapies such as vitamin supplementation have gained more attention, as they generally have few noticeable side effects apart from high-dose use (5). A number of studies suggested that migraine is correlated with nutrients, which may alleviate headache symptoms or reduce the prevalence of migraine (6–8). Therefore, exploring other potential diet nutrition related to migraine is necessary, which may aid in preventing or treating migraine.

Selenium is an essential micronutrient that is positively correlated with type 2 diabetes and negatively associated with the prevalence of coronary heart disease and cancer (9–11). Its deficiency has also been associated with neurological disorders and cognitive decline (12, 13). Selenium, mainly in the form of selenoprotein, inhibits oxidative damage in the brain and is involved in regulating inflammatory responses (14, 15). Nazıroğlu M et al. reported that oral selenium was administered to rat model of migraine, which protected against brain oxidative toxicity (16). Previous research indicated that the trinity of inflammation, mitochondrial dysfunction and oxidative stress might exert a role in the development of migraine (17, 18). However, there are no studies examining the association between dietary selenium intake and migraine in the general population.

Consequently, this study explored the relationship between dietary selenium intake and migraine in adults in a general population using data from the NHANES. We hypothesized that there was an inverse association between dietary consumption of selenium and migraine. Furthermore, subgroup analyses were conducted to assess possible effect modification of the relationship between dietary selenium intake and migraine. The restricted cubic spline (RCS) regression models were utilized to investigate the dose–response relationship.

## 2 Materials and methodology

### 2.1 Study design and population

The data analyzed in this cross-sectional study were obtained from the NHANES, administered by the Centers for Disease Control and Prevention. NHANES involved examinations, interviews, and questionnaires administered through home visits and mobile examination centers (MECs) to measure the health and nutritional history of the US population. The survey projects of NHANES were approved by the Ethics Review Committee of the National Center for Health Statistics.

**Figure 1:**
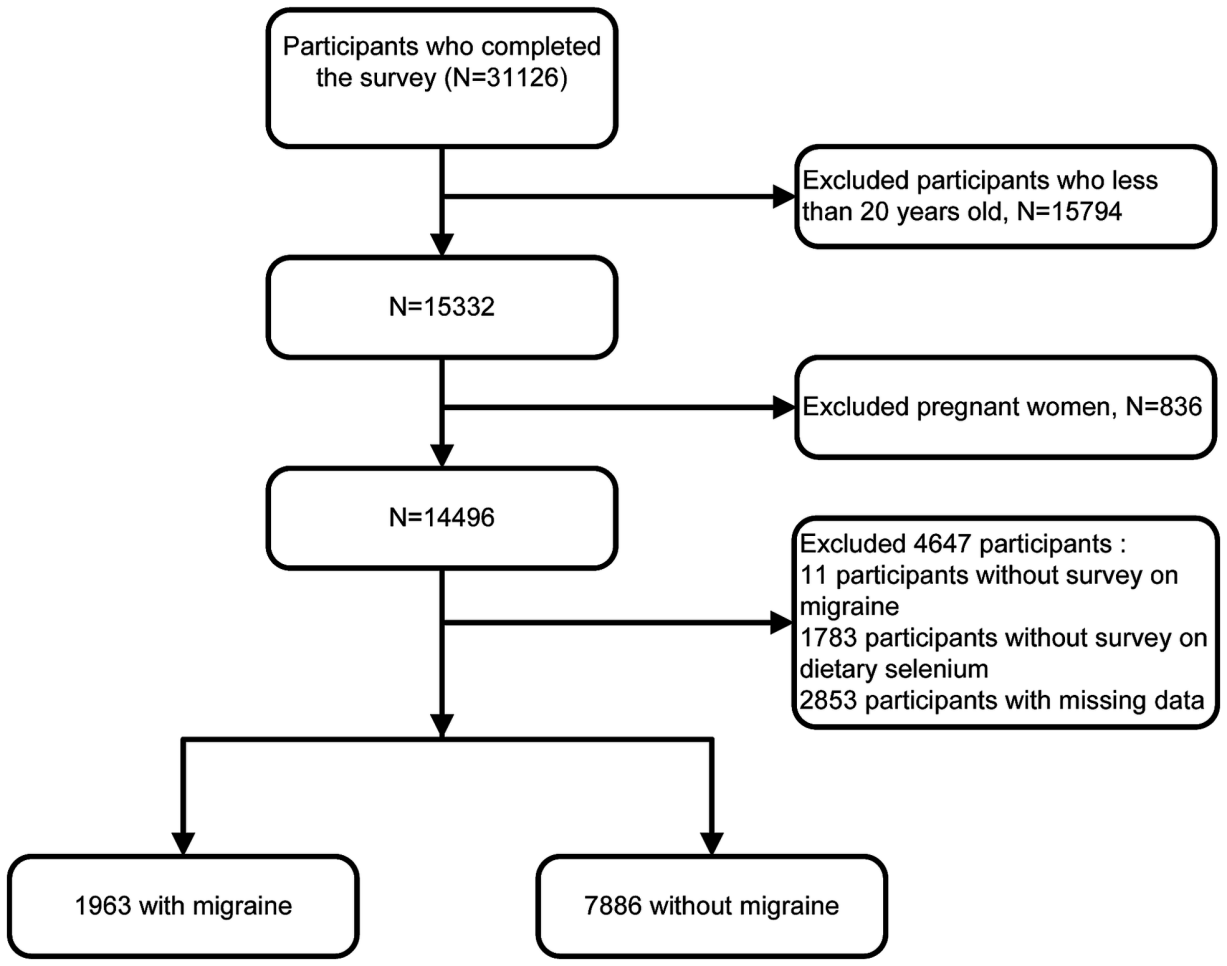
Inclusion and exclusion flow chart.

We conducted a cross-sectional study of American adults in the 1999–2004 NHANES survey, as this was the only cycle that included adult headache questionnaires. These data were combined for our analysis, which resulted in 31,126 participants. And our research was confined to adults aged 20 years or older. We eliminated pregnant women and participants with missing data, such as migraine questionnaires and dietary data. The final number of participants in this study was 9,849 (Figure 1).

### 2.2 Migraine classification

The outcome of migraine was assessed using self-reported data from the NHANES Miscellaneous Pain Questionnaire, where question MPQ090 asked, “In the past 3 months, did you have severe headaches or migraines?” Participants who answered ‘yes’ were classified as having migraine. And we can consider that the majority of participants with severe headache has migraine, which in accordance with the findings of the American Migraine Prevalence and Prevention (AMPP) study. The study showed that 17.4% of individuals reported “severe headache,” of which 11.8% met the International Headache Disorder Type II (ICHDII) criteria for migraine, 4.6% met the criteria for “probable migraine,” and only 1% were identified as “other severe headache” (19). The self-reported assessments of migraine are reasonably reliable in the general US population and have been utilized in previous epidemiological studies using data from NHANES (20, 21).

### 2.3 Dietary selenium assessment

Dietary intake data for selenium were extracted from the NHANES Dietary Interview - Total Nutrient Intake file. Nutrient data in this file were obtained by asking respondents about the types and amounts of food and beverages consumed in a 24-h period. Data from the 24-h dietary recall survey were acquired using the Computer-Assisted Dietary Interview System from 1999 to 2002 and the United States Department of Agriculture (USDA) Automated Multiple Pass Method (AMPM) from 2003 to 2004. A detailed description of the dietary survey methodology can be found in the NHANES Dietary Interviewer Procedures Manual. In the 2003–2004 NHANES cycle, a second dietary recall interview was conducted by telephone 3–10 days later, whereas in 1999–2002 dietary data were only available from a first 24-h recall interview conducted in person. Hence, only the first 24 h of dietary data from 2003 to 2004 were included in this study to keep consistency with 1999–2002.

### 2.4 Covariates

Various potential covariates were evaluated based on the published literature (22–25). The present study incorporated the following demographic covariates: age, sex, race, educational level (<high school, high school, >high school), marital status (married, living alone) and family income. The poverty income ratio (PIR) classified family income into three categories: low (PIR ≤ 1.3), medium (PIR > 1.3 ~ 3.5) and high (PIR >3.5). Data on protein, energy and carbohydrate intake were obtained from 24-h food recalls. Body mass index (BMI) was calculated using a standardized technique based on weight and height. C-reactive protein (CRP) was quantified by latex-enhanced nephelometry. Drinking and smoking status was classified as never, current and former, according to previous descriptions in the literature. History of stroke, hypertension, coronary heart disease or diabetes was assessed as self-reported physician diagnosis of stroke, hypertension, coronary heart disease or diabetes.

### 2.5 Statistical analyses

Statistical power calculations were not conducted before the study, and the sample size was all available data from NHANES. Dietary weights, primary sampling units and strata information were used in the statistical analysis. For the joint analysis of the NHANES 1999–2000 and 2001–2002 data, we used the 4-year dietary weight (WTDR4YR) set. For the 2003–2004 data, we used the dietary day-one sample weight (WTDRD1) set. According to the analysis guidelines available on the NHANES website, we calculated sample weights for 1999–2004 = 2/3 of the 1999–2002 weight or 1/3 of the 2003–2004 weight. Continuous variables are presented as sample-weighted mean standard error, while categorical variables are presented as sample-weighted percentages and frequencies. One-way analyses of variance (continuous variables) and chi-squared tests (categorical variables) were used to compare differences between groups. Multiple logistic regression models were used to estimate odd ratios (ORs) with 95% confidence intervals (CIs) of migraine at different quintiles of dietary selenium. The lowest quintile of intake was used as the reference group. Potential modifications of the relationship between dietary selenium and migraine were then estimated for the following variables: sex, age (20–50, >50 years). In addition to dietary intake, supplements are an important source. To evaluate the effect of selenium supplements on migraine, a sensitivity analysis was conducted on participants who completed the supplementation survey. For the sensitivity analysis, we excluded participants with extreme energy intakes (<500 or > 5,000 kcal per day).

Restricted cubic spline (RCS) regression was further used to investigate the non-linear relationship between dietary selenium intake and migraine. After adjusting for all potential confounders, we applied a two-piece-wise logistic regression model with smoothing to investigate the threshold for the association between dietary selenium intake and migraine. Inflection points were identified using the likelihood ratio test and the bootstrap resampling method. All statistical analyses were performed using R software (version 4.2.1). Two-sided *p*-values <0.05 were considered statistically significant differences.

## 3 Results

### 3.1 Baseline characteristic

Supplementary materials (Supplementary Table S1) showed the general characteristics of the included and excluded individuals. The baseline characteristics of the 9,849 included participants based on their selenium intake quintile are demonstrated in Table 1. A total of 1963 participants with migraine, while 7,886 participants without migraine. The average age of the individuals in this study was 46.56 (0.31) years, and 4,848 (50.67) individuals were female. Participants who consumed more selenium often tended to be younger, male, non-Hispanic White, had a higher educational level, had a high family income, current drinking, had a lower incidence of hypertension and stroke, had higher consumption of energy, proteins and carbohydrates, and lower serum CRP levels. As indicated in Table 2, the findings of the univariate analysis demonstrated that age, sex, race, education, family income, smoking status, diabetes, stroke, BMI, protein intake and CRP were related to migraine.

**Table 1:** Population characteristics by categories of dietary selenium intake.

Characteristic ^a^		Selenium intake, ug/d					
		Q1	Q2	Q3	Q4	Q5	
	Total	≤59.4	59.41–82.70	82.71–106	106.01–143.16	≥143.17	*p* value ^b^
No.	9,849	1973	1967	1972	1967	1970	
Age (years)	46.56 (0.31)	50.00 (0.49)	48.62 (0.52)	47.70 (0.62)	44.87 (0.46)	42.43 (0.46)	< 0.0001
Sex							< 0.0001
Male	5,001 (49.33)	633 (27.53)	769 (35.73)	963 (46.76)	1,161 (57.23)	1,475 (74.50)	
Female	4,848 (50.67)	1,340 (72.47)	1,198 (64.27)	1,009 (53.24)	806 (42.77)	495 (25.50)	
Marital status							0.12
Living alone	3,672 (35.48)	848 (38.60)	763 (36.12)	739 (35.51)	654 (32.79)	668 (34.87)	
Married	6,177 (64.52)	1,125 (61.40)	1,204 (63.88)	1,233 (64.49)	1,313 (67.21)	1,302 (65.13)	
Race							0.04
Non-Hispanic White	5,220 (74.51)	977 (71.84)	1,019 (74.26)	1,068 (75.97)	1,077 (75.21)	1,079 (75.01)	
Non-Hispanic Black	1791 (9.77)	433 (12.25)	345 (9.10)	357 (10.22)	315 (8.54)	341 (9.07)	
Mexican American	2,113 (6.63)	419 (5.92)	442 (6.49)	428 (6.40)	416 (6.69)	408 (7.49)	
Others	725 (9.09)	144 (9.99)	161 (10.14)	119 (7.41)	159 (9.56)	142 (8.43)	
Education level							< 0.0001
<High school	2,974 (18.67)	766 (26.10)	660 (19.53)	537 (15.79)	521 (16.69)	490 (16.17)	
High school	2,351 (25.80)	455 (25.78)	453 (25.56)	494 (28.05)	464 (23.58)	485 (26.14)	
>High school	4,524 (55.53)	752 (48.12)	854 (54.92)	941 (56.16)	982 (59.74)	995 (57.68)	
Family income							< 0.0001
Low	2,682 (20.90)	692 (29.06)	557 (21.19)	506 (20.60)	453 (16.32)	474 (18.46)	
Medium	3,824 (35.41)	745 (35.46)	803 (36.35)	796 (36.80)	764 (34.87)	716 (33.80)	
High	3,343 (43.70)	536 (35.49)	607 (42.46)	670 (42.60)	750 (48.81)	780 (47.74)	
Smoking status							0.15
Never	4,940 (49.70)	1,030 (50.40)	1,018 (48.95)	989 (51.46)	960 (49.62)	943 (48.28)	
Current	2,200 (24.60)	439 (26.76)	408 (24.76)	413 (22.86)	430 (22.55)	510 (26.21)	
Former	2,709 (25.69)	504 (22.84)	541 (26.28)	570 (25.68)	577 (27.83)	517 (25.52)	
Drinking							< 0.0001
Never	1,392 (12.62)	413 (18.47)	324 (13.85)	268 (13.11)	211 (9.77)	176 (9.77)	
Current	6,418 (70.38)	1,055 (60.30)	1,218 (68.50)	1,280 (69.28)	1,389 (69.28)	1,476 (76.76)	
Former	2039 (17.00)	505 (21.22)	425 (17.65)	424 (17.61)	367 (14.94)	318 (14.32)	
Diabetes	994 (6.95)	227 (8.21)	226 (7.57)	200 (6.88)	183 (5.75)	158 (6.56)	0.09
Hypertension	3,261 (28.53)	780 (34.69)	684 (28.77)	697 (29.63)	608 (26.32)	492 (24.29)	< 0.0001
Stroke	320 (2.41)	108 (4.33)	65 (2.46)	70 (2.41)	45 (1.63)	32 (1.50)	< 0.0001
Coronary heart disease	473 (4.05)	120 (5.01)	90 (4.05)	101 (4.19)	94 (4.54)	68 (2.64)	0.06
BMI (kg/m^2^)	28.10 (0.11)	28.11 (0.18)	27.67 (0.21)	28.11 (0.17)	28.23 (0.16)	28.34 (0.27)	0.23
Energy (kcal/day)	2206.54 (14.94)	1278.59 (16.26)	1727.15 (18.49)	2101.86 (24.82)	2451.62 (26.35)	3273.20 (36.42)	< 0.0001
Protein intake (g/day)	82.26 (0.67)	38.57 (0.51)	59.11 (0.55)	75.60 (0.56)	93.91 (0.78)	134.42 (1.07)	< 0.0001
Carbohydrate intake (g/day)	271.89 (2.26)	175.99 (2.62)	223.16 (3.52)	263.36 (3.62)	296.61 (4.07)	379.93 (5.76)	< 0.0001
C-reactive protein (mg/dl)	0.42 (0.01)	0.49 (0.02)	0.45 (0.02)	0.41 (0.02)	0.41 (0.01)	0.34 (0.02)	< 0.0001
Migraine	1963 (22.03)	446 (26.12)	394 (21.63)	396 (24.42)	367 (19.89)	360 (18.85)	< 0.001

a mean and percentages were weighted.

b *p value was calculated by weighted one-way analyses of variance for continuous variable and Chi-square test for categorical variables.*

BMI, body mass index.

**Table 2:** Relationship of covariates and migraine risk.

Variable	OR (95% CI)	*p* value
Age (years)	0.98 (0.97, 0.98)	<0.0001
Sex		
Female	1 (Reference)	
Male	0.49 (0.43, 0.56)	<0.0001
Marital status		
Living alone	1 (Reference)	
Married	0.96 (0.83, 1.11)	0.54
Race		
Non-Hispanic White	1 (Reference)	
Non-Hispanic Black	1.21 (1.00, 1.45)	0.04
Mexican American	1.16 (0.96, 1.41)	0.12
Others	1.21 (0.88, 1.66)	0.23
Education level		
<High school	1 (Reference)	
High school	0.87 (0.74, 1.03)	0.11
>High school	0.66 (0.56, 0.78)	<0.0001
Family income		
Low	1 (Reference)	
Medium	0.67 (0.55, 0.81)	<0.0001
High	0.46 (0.38, 0.57)	<0.0001
Smoking status		
Never	1 (Reference)	
Current	1.35 (1.17, 1.56)	<0.001
Former	0.79 (0.67, 0.94)	0.009
Drinking		
Never	1 (Reference)	
Current	0.87 (0.73, 1.04)	0.13
Former	1.05 (0.83, 1.32)	0.67
Diabetes		
No	1 (Reference)	
Yes	0.80 (0.65, 0.99)	0.04
Hypertension		
No	1 (Reference)	
Yes	1.06 (0.95, 1.19)	0.27
Stroke		
No	1 (Reference)	
Yes	1.48 (1.01, 2.17)	0.04
Coronary heart disease		
No	1 (Reference)	
Yes	0.71 (0.45, 1.12)	0.13
Body mass index (kg/m^2^)	1.02 (1.01, 1.03)	<0.0001
Energy (kcal/day)	1.00 (0.99, 1.01)	0.57
Protein intake (g/day)	0.99 (0.98, 0.99)	0.0021
Carbohydrate intake (g/day)	1.00 (0.99, 1.01)	0.16
C-reactive protein (mg/dl)	1.09 (1.02, 1.16)	0.008

OR, odds ratio, CI, confidence interval.

### 3.2 Association between dietary selenium intake and migraine

The results of the multi-factor logistic regression models were demonstrated in Table 3. In the crude, dietary selenium intakes were significantly negative relationship with risk of migraine. After adjustment for age and sex, the results of model1 were similar to the crude. Compared to participants in the lowest quintile (Q1) of dietary selenium Consumption (≤59.4 ug/day), the adjusted ORs for migraine in Q2 (59.41–82.70 ug/day), Q3 (82.71–106.00 ug/day), Q4 (106.01–143.16 ug/day), and Q5 (≥143.17 ug/day) were 0.82 (95% CI: 0.64–1.05, *p* = 0.11), 0.99 (95% CI: 0.77–1.26, *p* = 0.92), 0.74 (95% CI: 0.54–0.99, *p* = 0.04), and 0.68 (95% CI: 0.48–0.97, *p* = 0.03) in the model 2, respectively.

**Table 3:** Association between dietary selenium intake and migraine.

	OR (95% CI)						
Quintiles	No.	Crude	*p* value	Model 1	*p* value	Model 2	*p* value
Dietary selenium (ug/day)							
Q1 (≤59.4)	1973	1 (reference)		1 (reference)		1 (reference)	
Q2 (59.41–82.70)	1967	0.78 (0.63, 0.97)	0.03	0.79 (0.63, 0.99)	0.04	0.82 (0.64, 1.05)	0.11
Q3 (82.71–106)	1972	0.91 (0.76, 1.10)	0.34	0.99 (0.81, 1.21)	0.9	0.99 (0.77, 1.26)	0.92
Q4 (106.01–143.16)	1967	0.70 (0.57, 0.87)	0.0016	0.76 (0.60, 0.95)	0.02	0.74 (0.54, 0.99)	0.044
Q5 (≥143.17)	1970	0.66 (0.54, 0.80)	<0.0001	0.77 (0.63, 0.94)	0.01	0.68 (0.48, 0.97)	0.03
*p* for trend	-	<0.001	-	0.03	-	0.04	-

Crude was adjusted for nothing. Model 1 was adjusted for age and sex. Model 2 was adjusted for Model 1+ marital status, race, education level, family income, smoking status, drinking, hypertension, coronary heart disease, stroke, diabetes, body mass index, energy, protein intake, carbohydrate intake, and C-reactive protein.

OR odds ratio, CI confidence interval.

In the restricted cubic spline analyses (Supplementary Figure S1), we found a non-linear association between dietary selenium intake and migraine (*p* < 0.001), using a reference point of 93.7 ug/day. The OR values for migraine decreased with increasing dietary selenium intake.

### 3.3 Sensitivity analysis

Selenium supplementation data are available only for NHANES 2001–2004; not for 1999–2000. Of the 9,849 individuals, 3,968 completed the selenium supplementation survey and 2050 reported having used supplements. The majority of individuals (52.88%, 1084/2050) used 20 ug/day of selenium supplements, so dividing this into quintiles was not feasible. Therefore, the individuals were divided into two groups based on the median selenium supplementation. The ORs for migraine in group 2 (supplemental intake >20 ug/day) compared to group 1 (supplemental intake: ≤20 ug/day) was 1.06 (95% CI: 0.68–1.66, *p* = 0.76). Compared to individuals in Q1 for total selenium intake (≤72.54 ug/day), the adjusted ORs for migraine in Q2 (72.55–100 ug/day), Q3 (100.01–133.12 ug/day), Q4 (133.13–199.40 ug/day) and Q5 (≥199.41 ug/day) were 1.02 (95% CI: 0.69–1.51, *p* = 0.92), 0.79 (95% CI: 0.52–1.19, *p* = 0.21), 0.69 (95% CI: 0.48–0.98, *p* = 0.04), 0.82 (95% CI: 0.48–1.38, *p* = 0.39), respectively (Table 4).

**Table 4:** Association of migraine with selenium intake among participants.

	OR (95% CI)						
Selenium intake (ug/day)	No.	Crude	*p* value	Model 1	*p* value	Model 2	*p* value
Supplement (*n* = 2050)							
Q1 (≤20)	1,084	1 (reference)		1 (reference)		1 (reference)	
Q2 (>20)	966	1.10 (0.75, 1.62)	0.60	1.03 (0.70, 1.52)	0.86	1.06 (0.68, 1.66)	0.76
Total (*n* = 3,968)							
Q1 (≤72.54)	794	1 (reference)		1 (reference)		1 (reference)	
Q2 (72.55–100.00)	794	0.98 (0.69, 1.38)	0.90	1.03 (0.73, 1.45)	0.86	1.02 (0.69, 1.51)	0.92
Q3 (100.01–133.12)	793	0.75 (0.56, 1.01)	0.06	0.78 (0.57, 1.07)	0.12	0.79 (0.52, 1.19)	0.21
Q4 (133.13–199.40)	795	0.67 (0.51, 0.89)	0.008	0.71 (0.53, 0.96)	0.03	0.69 (0.48, 0.98)	0.04
Q5 (≥199.41)	792	0.77 (0.55, 1.07)	0.12	0.82 (0.58, 1.17)	0.26	0.82 (0.48, 1.38)	0.39
*p* for trend	-	0.10	-	0.23	-	0.47	-

Crude was adjusted for nothing. Model 1 was adjusted for age and sex. Model 2 was adjusted for Model 1+ marital status, race, education level, family income, smoking status, drinking, hypertension, coronary heart disease, stroke, diabetes, body mass index, energy, protein intake, carbohydrate intake, and C-reactive protein.

OR odds ratio, CI confidence interval.

After excluding the participants with extreme energy intake, 9,598 individuals remained, and the relationship between dietary selenium intake and migraine remained stable. Compared with participants with lowest selenium intake Q1 (≤60.00 ug/day), the adjusted OR values for dietary selenium intake and migraine in Q2 (60.01–82.78 ug/day), Q3 (82.79–105.52 ug/day), Q4 (105.53–140.90 ug/day) and Q5 (≥140.91 ug/day) were 0.87 (95% CI: 0.67–1.10, *p* = 0.23), 1.00 (95% CI: 0.79–1.26, *p* = 0.99), 0.81 (95% CI: 0.61–1.06, *p* = 0.12) and 0.69 (95% CI: 0.49–0.98, *p* = 0.04), respectively (Supplementary Table S2).

### 3.4 Subgroup analysis

**Figure 2:**
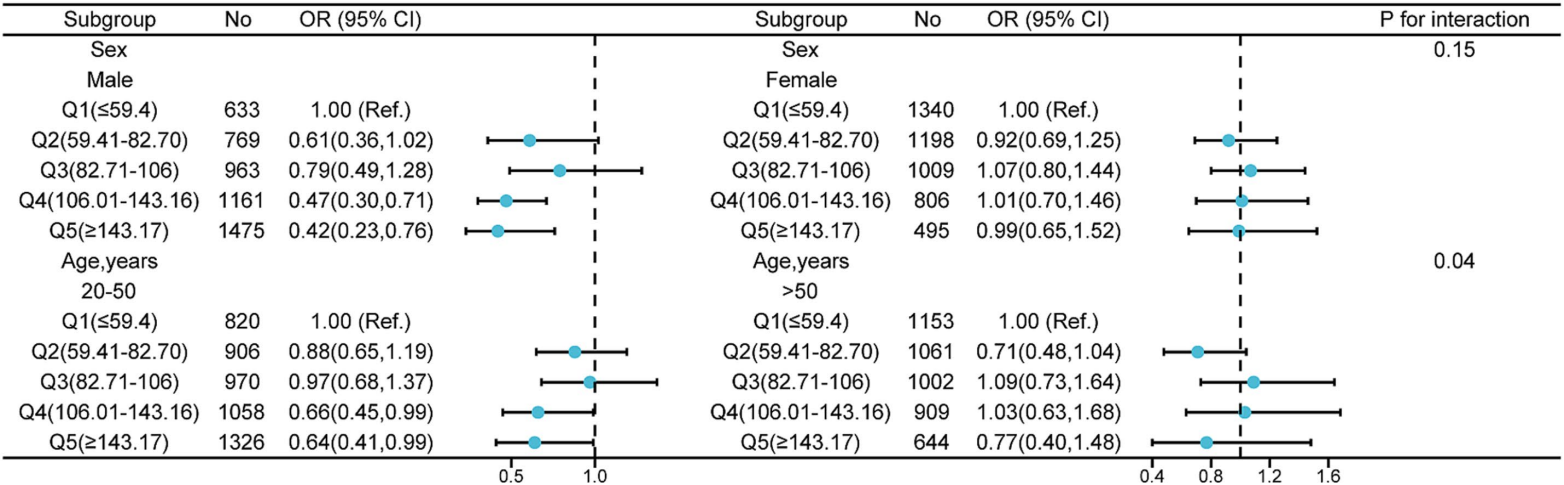
Effect of selenium intake on migraine in different subgroup (sex, age). Except the stratification variables themselves, each stratification factor was adjusted for all other variables (sex, age, marital status, race, education level, family income, smoking status, drinking, hypertension, coronary heart disease, stroke, diabetes, body mass index, energy, protein intake, carbohydrate intake, and C-reactive protein).

Subgroup analyses were performed in several subgroups to assess possible modifications on the effect of the association between dietary selenium intake and migraine. After stratification by sex and age, we observed a significant interaction between selenium intake and age (20–50 years) (Figure 2). The multivariate logistic regression model exhibited that the adjusted ORs for migraine were 0.88 (95% CI: 0.65–1.19), 0.97 (95% CI: 0.68–1.37), 0.66 (95% CI: 0.45–0.99) and 0.64 (95% CI: 0.41–0.99), respectively. Additionally, in the age > 50 years group, we observed that selenium intake was not related to the prevalence of migraine. And, we observed that no significant interactions in sex subgroups (Figure 2).

**Figure 3:**
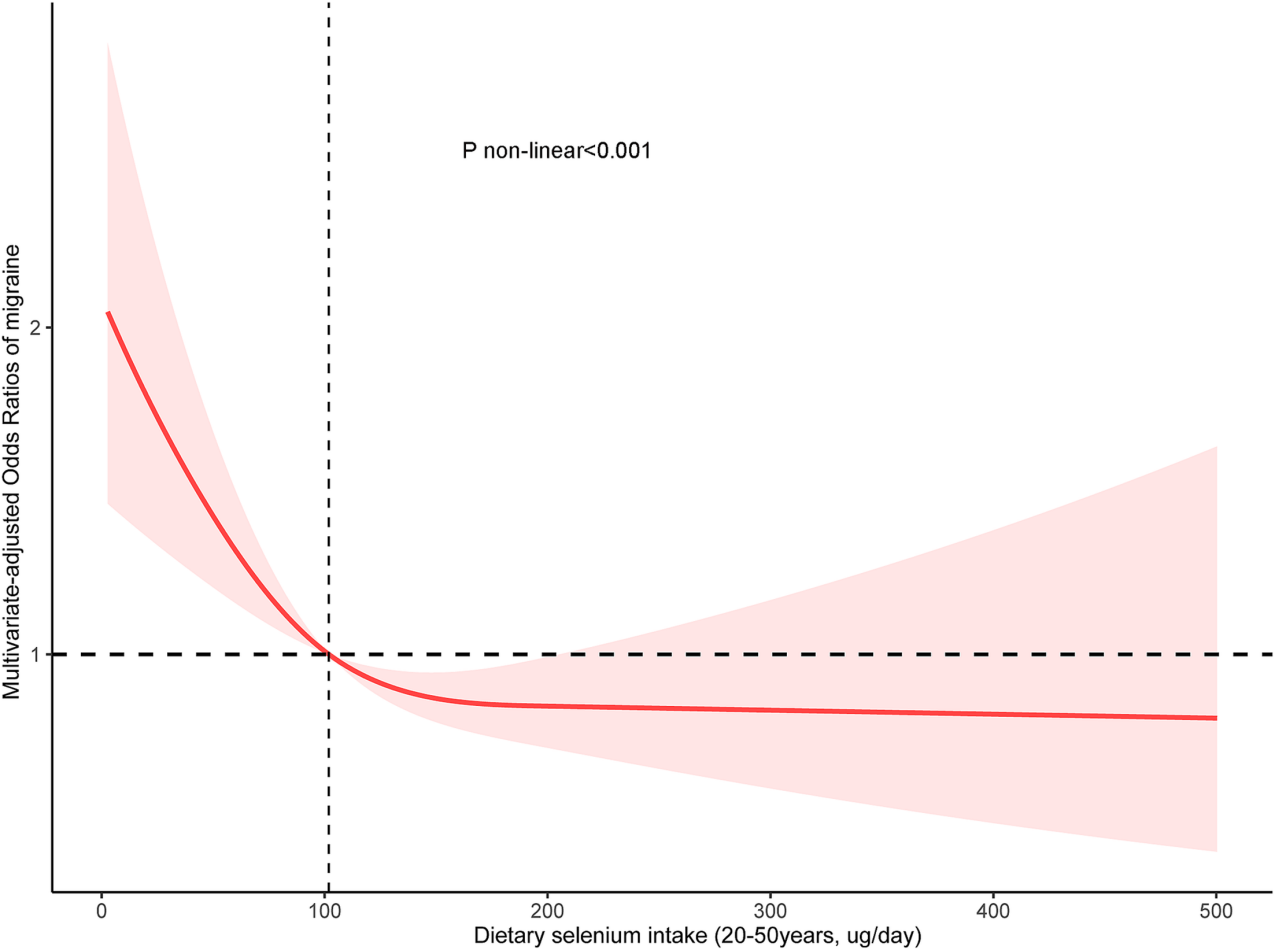
Association between dietary selenium intake and migraine odds ratio in the 20–50 years group. The model was adjusted for sex, marital status, race, education level, family income, smoking status, drinking, hypertension, coronary heart disease, stroke, diabetes, body mass index, energy, protein intake, carbohydrate intake, and C-reactive protein.

In the Figure 3, the restricted cubic spline also demonstrated that a L-shaped relationship between dietary selenium intake and migraine in the 20–50 years group (*p* < 0.001), using a reference point of 101.9 ug/day. The OR values for the association between selenium intake and migraine were decreased with increasing dietary selenium intake.

In the threshold analysis, the OR for migraine was 0.97 (95% CI: 0.94–0.98, *p* = 0.04) in individuals with a selenium intake of ≥101.9 ug/day (Table 5). It means that the risk of migraine is reduced by 3% with every 1 ug increase in selenium intake. There was no relationship between selenium intake and migraine when the selenium intake<101.9 ug/day (Table 5). It means that the risk of migraine no longer reduced with increasing selenium intake.

**Table 5:** Threshold effect analysis of the association of selenium intake with migraine in adults with 20–50 years.

Selenium intake ug/day	Adjust OR (95% CI)	*p*-value
<101.90	1.01 (0.99, 1.02)	0.64
≥101.90	0.97 (0.94, 0.98)	0.04
Log-likelihood ratio test		0.005

Fully adjusted for sex, marital status, race, education level, family income, smoking status, drinking, hypertension, coronary heart disease, stroke, diabetes, body mass index, energy, protein intake, carbohydrate intake, and C-reactive protein.

OR odds ratio, CI confidence interval.

## 4 Discussion

In the present study, we utilized NHANES 1999–2004 data that included 9,849 people to study the association between dietary selenium intake and migraine in American adults. Examination of dietary selenium intake revealed a negative association with migraine. The sensitivity analyses demonstrated a robust association between dietary selenium intake and migraine. And we found a non-linear association (*p* < 0.001) between dietary selenium intake and migraine in overall, and in the 20–50 years group (*p* < 0.001). In addition, age can modify the association between selenium intake and migraine. Especially, when selenium intake ≥101.9 ug /day, increased selenium intake was related to a lower risk of migraine in the 20–50 years group; this association was not observed in age > 50 years group.

The effect of selenium on migraine has only been reported in a few previous studies. A case–control study in Iran revealed that serum levels of selenium were significantly lower in migraine patients compared to healthy controls and that individuals with the lowest selenium levels were 11 times more likely to develop migraine than those with the highest selenium levels (26). A guideline from the Canadian Headache Society recommended moderate selenium supplementation to prevent migraines (27). In a double-blinded, randomized clinical trial involving 72 migraine patients, 200 μg/day of selenium supplementation for 12 weeks significantly reduced headache frequency and severity, and improved quality of life as measured by the Headache Impact Test-6 (HIT-6) (28). Selenium supplementation at this dose was generally well-tolerated, with no reported serious adverse effects in the clinical trial (28). While no significant adverse effects were observed in this study, excessive intake of selenium can lead to selenosis, characterized by symptoms such as hair loss, brittle nails, poor dental health, skin disorders, neurological disturbances and even paralysis (29). In our research, we found that the higher intake of selenium was negatively associated with migraine. Furthermore, we also performed sensitivity analyses to assess the relationship between selenium supplementation and migraine. There was no relationship between selenium supplementation and migraine; however, when dietary and supplemental selenium intake were combined, there was an inverse relationship between total selenium intake and migraine. Also, after excluding the participants with extreme energy intake, the robust association between them remained. Further researches are needed to verify these findings.

The findings of our cross-sectional study provide new insights. First of all, we offered epidemiological evidence of relationship between dietary selenium intake and migraine in a representative general population, compared to published studies with small samples. Secondly, we observed that there was an interaction between age and selenium intake (*p* for interaction = 0.04). A higher selenium intake level was related to a decreased risk of migraine in participants with 20–50 years. It is well known that the main factor affecting selenium levels in the body is dietary habits, especially the consumption of selenium-rich foods such as red meat, fish and dairy products (30). In the elderly (aged over 50), lower energy intake and consequently lower selenium intake (31). In addition, selenium-rich food sources are also protein-rich, which can be difficult for older individuals to purchase, prepare or consume (32, 33). As a result, differences in dietary patterns in different age groups lead to the possibility that selenium may have different effects on the onset of migraine. And our findings also showed that lower dietary intake of selenium tended to be older. Therefore, participants aged 20–50 years may have higher selenium absorption than participants over 50 years, which may be the reason why dietary selenium was associated with migraine only in participants aged 20–50 years. The negative association between selenium intake and migraine in individuals with 20–50 years was important to propose an individualized strategy for migraine prevention in adults. Based on the results of the threshold analysis, we recommend that adults should consume moderate amounts of selenium-rich foods.

Although the biological mechanism of the inverse relationship between selenium intake and migraine is still to be explored, our results are biologically plausible based on the available evidence. Various mechanisms have been discovered to explain these relationships. In recent years, the importance of oxidative stress in migraine has been considered. Oxidative stress is one of the molecular changes in the pathogenesis of migraine, and its role in migraine is crucial duo to it stimulates transient receptor potential subfamily A member 1 (TRPA1) ion channels on meningeal pain receptors and produces neuroinflammation (34–36). Selenium is incorporated, as a trace element, into selenoproteins, which are found in the brain (37). Selenoproteins, such as glutathione peroxidases (GPx), thioredoxin reductases (TrxRs), or selenoprotein P (SelP) have antioxidant activity; exert a key role in maintaining the physiological function of the nervous system (38). And selenium as an antioxidant showed protective effects in an animal model of glyceryl trinitrate-induced migraine (16). Selenium protects against nitroglycerin-induced brain oxidative toxicity by inhibiting free radicals and regulating microsomal membrane Ca(2+) -ATPase activity, as well as supporting antioxidant REDOX systems (16). In addition, some cytokines contribute to inflammation, pain threshold modulation and trigeminal nerve fiber sensitization, which may contribute to migraine attacks (39). However, selenium affects the synthesis of several inflammatory factors, such as IL-6, IL-8, IL-12 cyclooxygenase-2, IL-10 and TGF-*β* (40). Thus, these revealed that the antioxidant and anti-inflammatory properties of selenium may contribute to its beneficial effects on migraine.

There are some limitations that must be taken into account in this study. At first, this study is based on data from 1999 to 2004. However, it was used because NHANES did not collect data on migraine after 2004. While the data may not fully reflect current food consumption patterns, it is still highlighted in the most recent 2015–2020 Dietary Guidelines for Americans, that inadequate selenium intake remains prevalent in the US Population (41). And the identification of migraine was limited to a single question of migraine or severe headache within the past 3 months. In addition, there were no data on other characteristics of participants’ migraine, such as severity or the presence of aura and other symptoms. Nonetheless, previous studies have supported the consistency of this migraine assessment with criteria for migraine and possible migraine, as well as others who have published articles on migraine with this dataset on the basis of supporting data from the American Migraine Prevalence and Prevention Study (19, 25, 42). Thus, these data provide meaningful insight into the otherwise lack of epidemiologic data linking diet and migraine. Secondly, we cannot eliminate the effect of nonrandom missing data on the results because of baseline differences between included and excluded participants. Third, the number of individuals included a large group (≥20 years old). Also, this study was performed in United States population, and the results might not be extended to other populations. Finally, our study was a cross-sectional study, which meant that causal inferences cannot be made. Therefore, we will need to conduct the prospective cohort study to obtain further accurate evidence.

## 5 Conclusion

In conclusion, our study revealed an inverse relationship between selenium intake and migraine incidence, indicating that higher selenium intake is associated with a lower risk of migraines. Notably, this negative correlation was more pronounced among participants aged 20–50 years. However, the overall findings suggest that adequate selenium intake may be a beneficial dietary strategy for migraine prevention across different age groups. Future research should further explore the mechanisms underlying this association and investigate the potential impact of selenium on other age groups as well.

## Data Availability

Publicly available datasets were analyzed in this study. This data can be found at: https://www.cdc.gov/nchs/nhanes/index.htm.

## References

[1] GBD 2016 Headache Collaborators. Global, regional, and national burden of migraine and tension-type headache, 1990-2016: a systematic analysis for the global burden of disease study 2016. Lancet Neurol. (2018) 17:954–76. doi: 10.1016/S1474-4422(18)30322-3 30353868 PMC6191530

[2] Steiner TJ Stovner LJ Vos T. GBD 2015: migraine is the third cause of disability in under 50s. J Headache Pain. (2016) 17:104. doi: 10.1186/s10194-016-0699-5, PMID: 27844455 PMC5108738

[3] Burch R Rizzoli P Loder E. The prevalence and impact of migraine and severe headache in the United States: updated age, sex, and socioeconomic-specific estimates from government health surveys. Headache. (2021) 61:60–8. doi: 10.1111/head.14024, PMID: 33349955

[4] Whyte CA Tepper SJ. Adverse effects of medications commonly used in the treatment of migraine. Expert Rev Neurother. (2009) 9:1379–91. doi: 10.1586/ern.09.47, PMID: 19769452

[5] Abo-Elghiet F Elosaily H Hussein DK El-Shiekh RA A’aqoulah A Yousef EM. Bridging gaps in migraine management: a comprehensive review of conventional treatments, natural supplements, complementary therapies, and lifestyle modifications. Pharmaceuticals. (2025) 18:139. doi: 10.3390/ph18020139, PMID: 40005953 PMC11858087

[6] Sanders AE Shaikh SR Slade GD. Long-chain omega-3 fatty acids and headache in the U.S. population. Prostaglandins Leukot Essent Fatty Acids. (2018) 135:47–53. doi: 10.1016/j.plefa.2018.06.008, PMID: 30103932

[7] Zeng Z Li Y Lu S Huang W Di W. Efficacy of CoQ10 as supplementation for migraine: a meta-analysis. Acta Neurol Scand. (2019) 139:284–93. doi: 10.1111/ane.13051, PMID: 30428123

[8] Ramsden CE Zamora D Faurot KR MacIntosh B Horowitz M Keyes GS. Dietary alteration of n-3 and n-6 fatty acids for headache reduction in adults with migraine: randomized controlled trial. BMJ. (2021) 374:n1448. doi: 10.1136/bmj.n1448, PMID: 34526307 PMC8244542

[9] Wang X-L Yang T-B Wei J Lei G-H Zeng C. Association between serum selenium level and type 2 diabetes mellitus: a non-linear dose-response meta-analysis of observational studies. Nutr J. (2016) 15:48. doi: 10.1186/s12937-016-0169-6, PMID: 27142520 PMC4855440

[10] Flores-Mateo G Navas-Acien A Pastor-Barriuso R Guallar E. Selenium and coronary heart disease: a meta-analysis. Am J Clin Nutr. (2006) 84:762–73. doi: 10.1093/ajcn/84.4.762, PMID: 17023702 PMC1829306

[11] Cai X Wang C Yu W Fan W Wang S Shen N. Selenium exposure and Cancer risk: an updated Meta-analysis and Meta-regression. Sci Rep. (2016) 6:19213. doi: 10.1038/srep19213, PMID: 26786590 PMC4726178

[12] Akbaraly TN Hininger-Favier I Carrière I Arnaud J Gourlet V Roussel A-M. Plasma selenium over time and cognitive decline in the elderly. Epidemiology. (2007) 18:52–8. doi: 10.1097/01.ede.0000248202.83695.4e, PMID: 17130689

[13] Shahar A Patel KV Semba RD Bandinelli S Shahar DR Ferrucci L. Plasma selenium is positively related to performance in neurological tasks assessing coordination and motor speed. Mov Disord. (2010) 25:1909–15. doi: 10.1002/mds.23218, PMID: 20687175 PMC3270688

[14] Mostert V. Selenoprotein P: properties, functions, and regulation. Arch Biochem Biophys. (2000) 376:433–8. doi: 10.1006/abbi.2000.1735, PMID: 10775431

[15] Van Cauwenbergh R Robberecht H Van Vlaslaer V Deelstra H. Comparison of the serum selenium content of healthy adults living in the Antwerp region (Belgium) with recent literature data. J Trace Elem Med Biol. (2004) 18:99–112. doi: 10.1016/j.jtemb.2004.04.004, PMID: 15487770

[16] Nazıroğlu M Çelik Ö Uğuz AC Bütün A. Protective effects of riboflavin and selenium on brain microsomal Ca2+-ATPase and oxidative damage caused by glyceryl trinitrate in a rat headache model. Biol Trace Elem Res. (2015) 164:72–9. doi: 10.1007/s12011-014-0199-x, PMID: 25492827

[17] Borkum JM. Brain energy deficit as a source of oxidative stress in migraine: a molecular basis for migraine susceptibility. Neurochem Res. (2021) 46:1913–32. doi: 10.1007/s11064-021-03335-9, PMID: 33939061

[18] Erdener ŞE Kaya Z Dalkara T. Parenchymal neuroinflammatory signaling and dural neurogenic inflammation in migraine. J Headache Pain. (2021) 22:138. doi: 10.1186/s10194-021-01353-0, PMID: 34794382 PMC8600694

[19] Buse DC Loder EW Gorman JA Stewart WF Reed ML Fanning KM. Sex differences in the prevalence, symptoms, and associated features of migraine, probable migraine and other severe headache: results of the American migraine prevalence and prevention (AMPP) study. Headache. (2013) 53:1278–99. doi: 10.1111/head.12150, PMID: 23808666

[20] Zheng H Tian S Wu L Zhong X Liu M Yu X. Dietary zinc intake in relation to migraine among adults: a cross sectional study of NHANES 1999-2004. Nutr Neurosci. (2024) 27:667–76. doi: 10.1080/1028415X.2023.2243678, PMID: 37540169

[21] Li H Krall JR Frankenfeld C Slavin M. Nutritional intake of riboflavin (vitamin B2) and migraine: a cross-sectional analysis of the National Health and nutrition examination survey (NHANES) 2001-2004. Nutr Neurosci. (2023) 26:1068–77. doi: 10.1080/1028415X.2022.2126760, PMID: 36175363

[22] Liu H Wang Q Dong Z Yu S. Dietary zinc intake and migraine in adults: a cross-sectional analysis of the National Health and nutrition examination survey 1999-2004. Headache. (2023) 63:127–35. doi: 10.1111/head.14431, PMID: 36588459

[23] Meng S-H Wang M-X Kang L-X Fu J-M Zhou H-B Li X. Dietary intake of calcium and magnesium in relation to severe headache or migraine. Front Nutr. (2021) 8:653765. doi: 10.3389/fnut.2021.653765, PMID: 33748178 PMC7973018

[24] Li D Guo Y Xia M Zhang J Zang W. Dietary intake of thiamine and riboflavin in relation to severe headache or migraine: a cross-sectional survey. Headache. (2022) 62:1133–42. doi: 10.1111/head.14384, PMID: 36047917

[25] Slavin M Li H Khatri M Frankenfeld C. Dietary magnesium and migraine in adults: a cross-sectional analysis of the National Health and nutrition examination survey 2001-2004. Headache. (2021) 61:276–86. doi: 10.1111/head.14065, PMID: 33503279

[26] Talaie A Jafary H Faraji F Malekirad AA. The serum oxidative stress biomarkers and selenium levels in a Group of Migraine Patients Compared with healthy controls: a case-control study. Biol Trace Elem Res. (2022) 200:4250–5. doi: 10.1007/s12011-021-03024-2, PMID: 34985626

[27] Pringsheim T Davenport WJ Mackie G Worthington I Aubé M Christie SN. Canadian headache society guideline for migraine prophylaxis. Can J Neurol Sci. (2012) 39:S1–S59. PMID: 22683887

[28] Balali A Sadeghi O Khorvash F Rouhani MH Askari G. The effect of selenium supplementation on oxidative stress, clinical and physiological symptoms in patients with migraine: a double-blinded randomized clinical trial. Front Nutr. (2024) 11:1369373. doi: 10.3389/fnut.2024.1369373, PMID: 38757125 PMC11096528

[29] Rayman MP. Selenium and human health. Lancet. (2012) 379:1256–68. doi: 10.1016/S0140-6736(11)61452-9, PMID: 22381456

[30] Combs GF. Selenium in global food systems. Br J Nutr. (2001) 85:517–47. doi: 10.1079/bjn2000280, PMID: 11348568

[31] González S Huerta JM Fernández S Patterson EM Lasheras C. Food intake and serum selenium concentration in elderly people. Ann Nutr Metab. (2006) 50:126–31. doi: 10.1159/000090633, PMID: 16391467

[32] Rayman MP. Food-chain selenium and human health: emphasis on intake. Br J Nutr. (2008) 100:254–68. doi: 10.1017/S0007114508939830, PMID: 18346308

[33] Coelho-Júnior HJ Rodrigues B Uchida M Marzetti E. Low protein intake is associated with frailty in older adults: a systematic review and Meta-analysis of observational studies. Nutrients. (2018) 10:1334. doi: 10.3390/nu10091334, PMID: 30235893 PMC6165078

[34] Borkum JM. The migraine attack as a homeostatic, neuroprotective response to brain oxidative stress: preliminary evidence for a theory. Headache. (2018) 58:118–35. doi: 10.1111/head.13214, PMID: 29034461

[35] Gumusyayla S Vural G Bektas H Neselioglu S Deniz O Erel O. A novel oxidative stress marker in migraine patients: dynamic thiol-disulphide homeostasis. Neurol Sci. (2016) 37:1311–7. doi: 10.1007/s10072-016-2592-z, PMID: 27142446

[36] Mohammadi H Talebi S Ghavami A Rafiei M Sharifi S Faghihimani Z. Effects of zinc supplementation on inflammatory biomarkers and oxidative stress in adults: a systematic review and meta-analysis of randomized controlled trials. J Trace Elem Med Biol. (2021) 68:126857. doi: 10.1016/j.jtemb.2021.126857, PMID: 34560424

[37] Chen J Berry MJ. Selenium and selenoproteins in the brain and brain diseases. J Neurochem. (2003) 86:1–12. doi: 10.1046/j.1471-4159.2003.01854.x, PMID: 12807419

[38] Xiong S Markesbery WR Shao C Lovell MA. Seleno-L-methionine protects against beta-amyloid and iron/hydrogen peroxide-mediated neuron death. Antioxid Redox Signal. (2007) 9:457–67. doi: 10.1089/ars.2006.1363, PMID: 17280487

[39] Biscetti L Cresta E Cupini LM Calabresi P Sarchielli P. The putative role of neuroinflammation in the complex pathophysiology of migraine: From bench to bedside. Neurobiology of Disease. (2023) 180:106072. doi: 10.1016/j.nbd.2023.106072 PMID: 36907522

[40] Li S Zhao Q Zhang K Sun W Jia X Yang Y. Se deficiency induces renal pathological changes by regulating selenoprotein expression, disrupting redox balance, and activating inflammation. Metallomics. (2020) 12:1576–84. doi: 10.1039/d0mt00165a, PMID: 32869810

[41] U.S. Department of Health and Human Services and U.S. Department of Agriculture. 2015-2020 dietary guidelines for Americans. 8th ed. Washington, DC: U.S. Government Printing Office, U.S. Department of Agriculture and U.S. Department of Health and Human Services (2015).

[42] Pogoda JM Gross NB Arakaki X Fonteh AN Cowan RP Harrington MG. Severe headache or migraine history is inversely correlated with dietary sodium intake: NHANES 1999-2004. Headache. (2016) 56:688–98. doi: 10.1111/head.12792, PMID: 27016121 PMC4836999

