# Supplementary material for: Interaction between dietary selenium intake and age on severe headache or migraine in the United States: a population-based study
Source: Front Nutr. 2025 Mar 25;12:1537151. doi: 10.3389/fnut.2025.1537151 (PMC11975585; doi:10.3389/fnut.2025.1537151)
Supplement: Supplementary file 1 [file Table_1.DOCX]

**Table S1** Basic characteristics of excluded and included participants

|  | Excluded population | Included population | *P* value ^b^ |
| --- | --- | --- | --- |
| Number of subjects ^a^ (%) | 21277(68.4%) | 9849 (31.6%) |  |
| Sex (%) |  |  | <0.001 |
| Male | 10183 (47.9) | 5001 (50.8) |  |
| Female | 11094 (52.1) | 4848 (49.2) |  |
| Age(year) | 19.92 (21.39) | 50.74 (18.46) | <0.001 |
| Race (%) |  |  |  |
| Non-Hispanic White | 6886 (32.4) | 5220 (53.0) | <0.001 |
| Non-Hispanic Black | 5781 (27.2) | 1791 (18.2) |  |
| Mexican American | 6575 (30.9) | 2113 (21.5) |  |
| Others | 2035 (9.6) | 725 (7.4) |  |
| Marital status (%) |  |  |  |
| Living alone | 7209 (70.4) | 3672 (37.3) | <0.001 |
| Married | 3024 (29.6) | 6177 (62.7) |  |
| Education (%) |  |  |  |
| <High school | 11716 (73.2) | 2974 (30.2) | <0.001 |
| High school | 1873 (11.7) | 2351 (23.9) |  |
| >High school | 2422 (15.1) | 4524 (45.9) |  |
| Family income (%) |  |  |  |
| Low | 8098 (44.0) | 2682 (27.2) | <0.001 |
| Medium | 6493 (35.3) | 3824 (38.8) |  |
| High | 3822 (20.8) | 3343 (33.9) |  |
| Smoking status |  |  |  |
| Never | 2965 (54.4) | 4940 (50.2) | <0.001 |
| Current | 1103 (20.2) | 2200 (22.3) |  |
| Former | 1380 (25.3) | 2709 (27.5) |  |
| Drinking |  |  |  |
| Never | 602 (18.6) | 1392 (14.1) | <0.001 |
| Current | 1910 (59.1) | 6418 (65.2) |  |
| Former | 719 (22.3) | 2039 (20.7) |  |
| Diabetes | 582 (2.9) | 994 (10.1) | <0.001 |
| Hypertension | 1753 (20.1) | 3261 (33.1) | <0.001 |
| Stroke | 286 (5.2) | 320 (3.2) | <0.001 |
| Coronary heart disease | 234 (4.3) | 473 (4.8) | 0.22 |
| BMI (kg/m^2^) | 22.78 (6.71) | 28.38 (6.21) | <0.001 |
| Energy (kcal/day) | 1978.27 (982.91) | 2121.86 (1023.94) | <0.001 |
| Protein intake (g/day) | 68.81 (39.82) | 79.73 (41.98) | <0.001 |
| Carbohydrate intake(g/day) | 263.98 (136.02) | 262.31 (134.39) | 0.33 |
| C-reactive protein (mg/dl) | 0.28 (0.70) | 0.47 (0.93) | <0.001 |
| Migraine | 1082 (19.8) | 1963 (19.9) | 0.84 |

^a^ mean and percentages are unweighted

^b^ p value was calculated by independent t-test for continuous variable and Chi-square test for categorical variables

BMI body mass index
