# Supplementary material for: Interaction between dietary selenium intake and age on severe headache or migraine in the United States: a population-based study
Source: Front Nutr. 2025 Mar 25;12:1537151. doi: 10.3389/fnut.2025.1537151 (PMC11975585; doi:10.3389/fnut.2025.1537151)
Supplement: Supplementary file 2 [file Table_2.DOCX]

**Table S2** Association between dietary selenium intake and migraine in participants with extreme energy intake was not included.

|  | |  |  |  | **OR (95% CI)** |  |  |  |
| --- | --- | --- | --- | --- | --- | --- | --- | --- |
| **Quintiles** | **No.** | | **Crude** | ***P* value** | **Model 2** | ***P* value** | **Model 3** | ***P* value** |
| **Selenium intake (ug/day)** |  | |  |  |  |  |  |  |
| Q1(≤60.00) | 1922 | | 1.00 (reference) |  | 1.00 (reference) |  | 1.00 (reference) |  |
| Q2(60.01-82.78) | 1917 | | 0.83(0.67,1.03) | 0.09 | 0.80(0.64,1.00) | 0.05 | 0.87(0.67,1.10) | 0.23 |
| Q3(82.79-105.52) | 1920 | | 0.97(0.79,1.18) | 0.73 | 0.92(0.75,1.13) | 0.40 | 1.00(0.79,1.26) | 0.99 |
| Q4(105.53- 140.90) | 1923 | | 0.76(0.62,0.93) | 0.01 | 0.68(0.55,0.83) | <0.001 | 0.81(0.61,1.06) | 0.12 |
| Q5(≥140.91) | 1916 | | 0.67(0.54,0.84) | <0.001 | 0.58(0.46,0.71) | <0.0001 | 0.69(0.49,0.98) | 0.04 |
| *P* for trend | - | | <0.001 | - | <0.0001 | - | 0.06 | - |

Crude was adjusted by nothing. Model 1 was adjusted for age and sex. Model 2 was adjusted for Model 1+marital status, race, education level, family income, smoking status, drinking, hypertension, coronary heart disease, stroke, diabetes, body mass index, energy, protein intake, carbohydrate intake, and C-reactive protein.

OR odds ratio, CI confidence interval.
