# Supplementary material for: Interaction between dietary selenium intake and age on severe headache or migraine in the United States: a population-based study
Source: Front Nutr. 2025 Mar 25;12:1537151. doi: 10.3389/fnut.2025.1537151 (PMC11975585; doi:10.3389/fnut.2025.1537151)
Supplement: Supplementary file 3 [file Image_1.pdf]

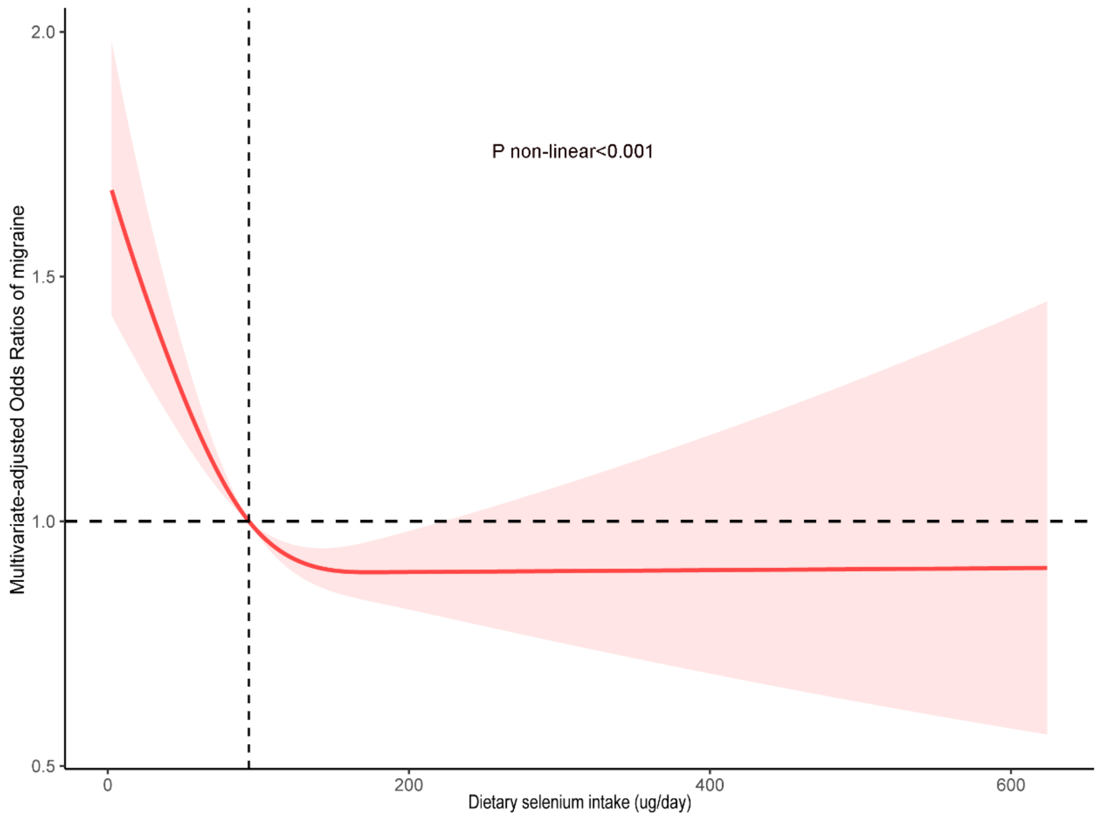

Figure S1 Relationship between dietary selenium intake and migraine odds ratio. The model was adjusted for sex, age, marital status, race, education level, family income, smoking status, drinking, hypertension, coronary heart disease, stroke, diabetes, body mass index, energy, protein intake, carbohydrate intake, and C-reactive protein
